# How fast can glucose be infused in the perioperative setting?

**DOI:** 10.1186/s13741-015-0027-7

**Published:** 2016-01-11

**Authors:** Robert G. Hahn

**Affiliations:** Research Unit at Södertälje Hospital, 152 86 Södertälje, Sweden

**Keywords:** Blood glucose, Metabolism glucose, Pharmacokinetics, Hyperglycemia

## Abstract

**Background:**

How the initial infusion rate of glucose solution should be set to avoid hyperglycemia in the perioperative setting is unclear.

**Methods:**

Computer simulations were performed based on data from seven studies where the kinetics of glucose was calculated using a one-compartment model. Glucose had been infused intravenously on 44 occasions to volunteers and on 256 occasions to surgical patients at various stages of the perioperative process. The rates that yield plasma glucose concentrations of 7, 9, and 12 mmol/l were calculated and standardized to a 5 % glucose solution infused in a subject weighing 70 kg.

**Results:**

The lowest infusion rates were found during surgery and the first hours after surgery. No more than 0.5 ml/min of glucose 5 % could be infused if plasma glucose above 7 mmol/l was not allowed, while 2 ml/min maintained a steady state concentration of 9 mmol/l. Intermediate infusion rates could be used in the preoperative period and 1–2 days after moderate-sized surgery (e.g., hysterectomy or hip replacement). Here, the half-lives averaged 30 min, which means that plasma glucose would rise by another 25 % if a control sample is taken 1 h after a continuous infusion is initiated. The highest infusion rates were found in non-surgical volunteers, where 8 ml/min could be infused before 9 mmol/l was reached.

**Conclusions:**

Computer simulations suggested that rates of infusion of glucose should be reduced by 50 % in the perioperative period and a further 50 % on the day of surgery in order to avoid hyperglycemia.

## Background

Intravenous glucose is the hallmark of maintenance fluid therapy to prevent starvation and provide free water for intracellular hydration. However, practices differ regarding its use in the perioperative period. Oral intake is the recommended type of carbohydrate administration in routine patients, but various reasons may call for the use of intravenous glucose both before and after surgery. Providing intravenous glucose carries the risk of inducing hyperglycemia, which promotes postoperative infection (Hahn and Hahn 2011; Sieber et al. 1987; Kwon et al. 2013; Frisch et al. 2010; Hanazaki et al. 2009; Lipshutz and Gropper 2009) and osmotic diuresis (Doze and White 1987). Very high glucose concentrations lead to more pronounced cerebral damage in the event of cardiac arrest (Myers and Yamaguchi 1977; Siemkowicz 1985).

Infusion rates that provide effective fluid and nutritional support therapy while avoiding hyperglycemia might be difficult to determine in the perioperative setting, as glucose turnover becomes impaired as part of the trauma response (Ljunggren et al. 2014a). Plasma glucose should be measured to guide adjustments of the infusion rate, but the point at which the check best reflects the risk of hyperglycemia is unclear to most clinicians.

The aim of the present work was to predict how fast glucose can be infused to reach specific concentrations of plasma glucose at defined points in time during the perioperative period in non-diabetic patients. These rates may serve as approximations of how initial infusion rates should be set, depending on the level to which the plasma glucose can be allowed to rise.

The hypothesis was that infusion rates should be reduced by at least 50 % as compared to healthy humans during and after surgery. To indicate the degree by which the rates should be modified, computer simulations were performed based on kinetic data from seven previous studies of glucose administration performed at various stages of the perioperative period (Sjöstrand and Hahn 2003; Hahn et al. 2011; Hahn et al. 2013; Ljunggren and Hahn 2012; Sjöstrand and Hahn 2004; Sicardi Salomón et al. 2006; Strandberg and Hahn 2005).

## Methods

This study is based on 321 infusion experiments, performed between 2002 and 2012, in which glucose was administered by intravenous infusion. The subjects were 26 volunteers and 161 patients in various stages of the perioperative process. All subjects gave their consent for participation after being informed about the purpose of the study. The results have been published in seven previous reports (Sjöstrand and Hahn 2003; Hahn et al. 2011; Hahn et al. 2013; Ljunggren and Hahn 2012; Sjöstrand and Hahn 2004; Sicardi Salomón et al. 2006; Strandberg and Hahn 2005). Four of them excluded patients with any disease (Sjöstrand and Hahn 2003; Hahn et al. 2011; Sjöstrand and Hahn 2004; Strandberg and Hahn 2005), and three excluded patients with disease of importance to glucose and fluid turnover (Hahn et al. 2013; Ljunggren and Hahn 2012; Sicardi Salomón et al. 2006).

### Ethics

The appropriate Ethics Committee approved the protocol for each of the studies. These were the Ethics Committee of Huddinge Hospital (Sjöstrand and Hahn 2003; Sjöstrand and Hahn 2004; Strandberg and Hahn 2005) and, later, the Regional Ethics Committee of Stockholm (Hahn et al. 2011; Hahn et al. 2013; Ljunggren and Hahn 2012; Sicardi Salomón et al. 2006). The approval numbers and the dates of decision were 258/00 (June 5, 2000) (Sjöstrand and Hahn 2003), 2007/1670-31 (January 30, 2008) (Hahn et al. 2011), 2011/1141-31/3 (September 28, 2011) (Hahn et al. 2013), 2008/1691-31/4 (September 28, 2011) (Ljunggren and Hahn 2012), 429/97 (January 12, 1998) (Sjöstrand and Hahn 2004), 19/03 (February 11, 2003) (Sicardi Salomón et al. 2006) and 34/99 (March 29, 1999) (Strandberg and Hahn 2005). The chairpersons were Lennart Kaijser (Sjöstrand and Hahn 2003; Sjöstrand and Hahn 2004; Strandberg and Hahn 2005), Hans Glaumann (Sicardi Salomón et al. 2006), Olof Forssberg (Hahn et al. 2011), Håkan Julius (Hahn et al. 2013), and Annika Marcus (Ljunggren and Hahn 2012).

### Procedures and measurements

All subjects were in the fasting state, which means that no food or sugar-containing beverages had been ingested for at least 4 h. Experiments started in the morning, and the only fluid given was a solution containing glucose 2.5 % (Sjöstrand and Hahn 2003; Sjöstrand and Hahn 2004; Sicardi Salomón et al. 2006; Strandberg and Hahn 2005) or 30 % (Hahn et al. 2011; Hahn et al. 2013; Ljunggren and Hahn 2012). The fluid with the low glucose concentration was buffered and half-isotonic with regard to electrolytes (sodium 70, chloride 45, and acetate 25 mmol/l), while the other solution contained only glucose. Infusion times varied between 1 and 80 min, and the total amount of glucose was usually between 200 and 300 mg/kg (Table 1). General anesthesia was used in the patients where the glucose kinetics was studied during ongoing surgery. Pain relief was enhanced by thoracic epidural anesthesia in nine of these patients who underwent major abdominal surgery.

**Table 1:** Data on the groups used for the simulations. The results are given as the mean (SD)

Study group	Experiments (*N*)	Age (years)	Body weight (kg)	Female/males	Glucose load (g/kg)	Plasma glucose baseline (mmol/l)	Plasma insulin baseline (pmol/l)	^10^log HOMA-IR^a^	*V*_d_/BW (ml/kg)	CL*/*BW (ml/(kg min))	Half-life^b^ (min)	Reference
Healthy volunteers	44	29 (7)	73 (14)	8/18^c^	0.25–0.62	5.0 (0.4)	35 (23)	2.15 (0.33)	164 (74)	8.7 (3.4)	15 (8)	Sjöstrand and Hahn (2003); Hahn et al. (2011)
1 day before hip replacement surgery	82	68 (9)	82 (15)	54/28	0.20–0.30	5.2 (0.7)	62 (50)	2.38 (0.35)	164 (29)	4.7 (1.8)	28 (11)	Hahn et al. (2013); Ljunggren and Hahn (2012)
During hernia surgery	9	37 (16)	80 (11)	0/9	≈0.20	5.7 (0.4)	–	–	152 (24)	1.6 (0.3)	66 (13)	Sicardi Salomón et al. (2006)^d^
During laparoscopic cholecystectomy	20	40 (8)	75 (10)	14/6	0.25–0.47	5.0 (0.6)	45 (26)	2.29 (0.25)	121 (19)	2.8 (0.7)	33 (12)	Sjöstrand and Hahn (2004); Sicardi Salomón et al. (2006)^d^
During open abdominal surgery	9	69 (6)	63 (11)	4/5	≈0.20	5.7 (1.1)	–	–	190 (34)	1.5 (0.7)	110 (67)	Sicardi Salomón et al. (2006)^d^
2–3 h after hip replacement surgery	60	68 (9)	83 (15)	41/9	0.20	5.9 (1.1)	47 (28)	2.35 (0.34)	174 (34)	3.3 (0.9)	38 (11)	Ljunggren and Hahn (2012)
1 day after hip replacement surgery	82	68 (9)	82 (15)	41/19	0.20–0.30	6.3 (0.8)	66 (44)	2.55 (0.27)	170 (31)	3.7 (1.3)	35 (13)	Hahn et al. (2013); Ljunggren and Hahn (2012)
2 days after hysterectomy	15	50 (5)	70 (9)	15/0	0.31	6.2 (0.7)	35 (16)	2.28 (0.25)	147 (41)	6.1 (1.2)	17 (5)	Strandberg and Hahn (2005)

^a^The ^10^log of (P-glucose × P-insulin). For crude HOMA-IR, the product should be divided by 156 to correct for units (22.5 if insulin is reported in mU l^−1^) where 1 = normal

^b^The half-life was obtained as 0.693 *V*_d_/CL

^c^Six males underwent four experiments each

^d^The study divided the kinetics into infusion and postinfusion phase. The kinetics from the infusion was used here

Plasma glucose concentrations in venous blood were measured on 7–20 occasions, using the glucose oxidase method. Duplicate samples were usually drawn at baseline and ensured a coefficient of variation of 1.2–1.5 %.

### Calculations

Kinetic analyses and simulations were made according to a one-compartment model implemented in MATLAB 4.2 (MathWorks Inc., Natick, MA). Simulations employed mean and individual data from the seven studies to predict the infusion rates required to reach and to maintain each one of three predetermined concentrations of plasma glucose (7, 9, and 12 mmol/l). The infusion rates required to reach these targets within 30 min were also calculated. The equations used are specified in the Appendix.

Insulin resistance was estimated by the Homeostatic Model Assessment of Insulin Resistance (HOMA-IR), which was the product of the plasma concentrations of glucose (mmol/l) and insulin (pmol/) just before the infusion started (Ljunggren and Hahn 2012). These data were available in all studied except one (Sicardi Salomón et al. 2006). No correction for units was made, but HOMA-IR was ^10^log-transformed to obtain a linear correlation with the hyperinsulinemic glucose clamp (Borai et al. 2007; Ljunggren et al. 2014b).

Data are presented as the means (standard deviation).

## Results

The characteristics of the studied cohorts are presented in Table 1. Twenty-one experiments were excluded because of incomplete data, leaving 300 experiments to be included in the final analysis.

**Fig. 1:**
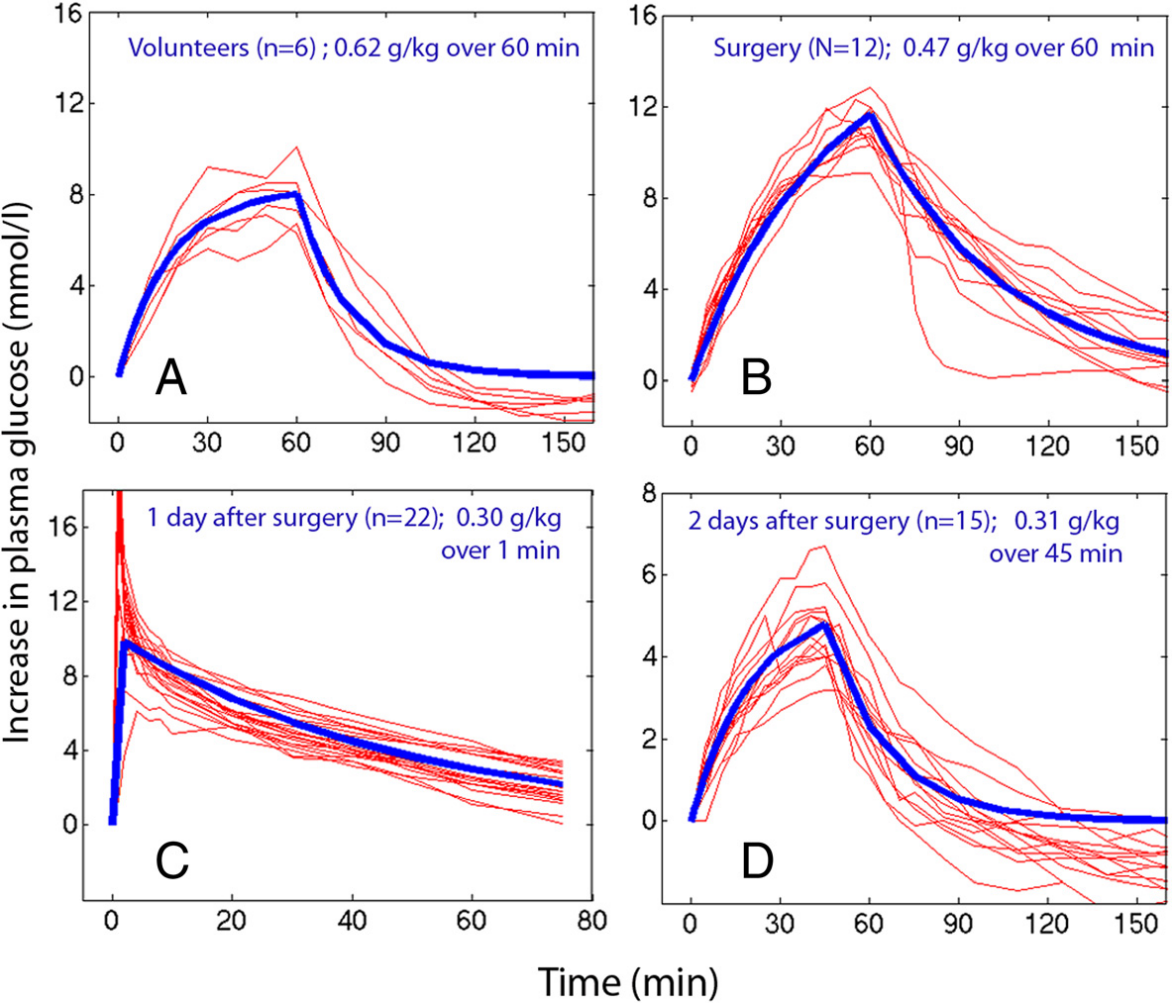
Examples of plasma glucose-time curves. Individual subjects (*thin lines*) and the modeled curve based on the mean values of the kinetic parameters *V*_d_ and CL (*thick line*). **a** is based on data from Sjöstrand and Hahn (2003), **b** from Sicardi Salomón et al. (2006), **c** original figure from Hahn et al. (2013), and **d** re-written based on data from Strandberg and Hahn (2005)

Plasma glucose from representative series of experiments is shown in Fig. 1.

The volume of distribution (*V*_d_), clearance (CL), and HOMA-IR for each study group is shown in Table 1.

**Fig. 2:**
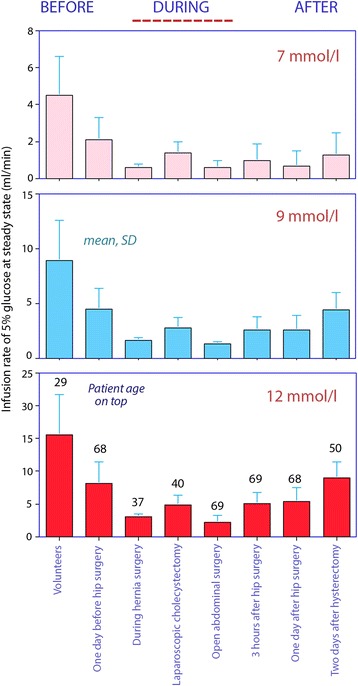
Rates of infusion of glucose 5 % required to maintain plasma glucose concentrations (steady state) of 7, 9, and 12 mmol/l during the perioperative period, standardized to a body weight of 70 kg. The mean age of each group is shown on *top* of the *bottom columns*

The infusion rates of glucose 5 % predicted to reach and maintain steady state levels of 7, 9, and 12 mmol/l in a subject weighing 70 kg based on each experiment separately are illustrated graphically in Fig. 2 and also given in Table 2, left.

**Table 2:** Rates of infusion of glucose 5 % required for reaching various predetermined target steady-state concentrations of plasma glucose (left) and rates of infusion required to reach the target within 30 min (right)

Study group	Infusion rate (ml/min) at steady state			Infusion rate (ml/min) to reach target after 30 min		
	7 mmol/l	9 mmol/l	12 mmol/l	7 mmol/l	9 mmol/l	12 mmol/l
Healthy volunteers	4.5 (2.1)	8.9 (3.7)	15.5 (6.2)	5.7 (2.6)	11.3 (4.3)	19.7 (7.1)
1 day before hip replacement surgery	2.1 (1.2)	4.5 (1,9)	8.1 (3.3)	3.7 (1.5)	7.8 (2.0)	14.0 (2.8)
During hernia surgery	0.6 (0.2)	1.6 (0.3)	3.0 (0.5)	2.3 (0.9)	5.7 (1.4)	10.9 (2.3)
During laparoscopic cholecystectomy	1.4 (0.6)	2.8 (0.9)	4.9 (1.4)	2.7 (1.3)	5.6 (1.5)	9.9 (2.1)
During open abdominal surgery	0.6 (0.4)	1.3 (0.2)	2.2 (1.1)	2.3 (1.6)	5.5 (1.8)	10.3 (2.5)
2–3 h after hip replacement surgery	1.0 (0.9)	2.6 (1.2)	5.1 (1.7)	2.1 (2.2)	6.0 (2.4)	11.7 (2.9)
1 day after hip replacement surgery	0.7 (0.8)	2.6 (1.3)	5.4 (2.1)	1.4 (1.6)	5.2 (1.9)	11.1 (2.6)
2 days after hysterectomy	1.3 (1.2)	4.4 (1.6)	9.0 (2.4)	1.8 (1.5)	6.1 (1.7)	12.6 (2.5)

All rates are adapted for subjects weighing 70 kg. Data are the mean (SD). Calculations begin with the actual baseline plasma glucose level of each subject

**Fig. 3:**
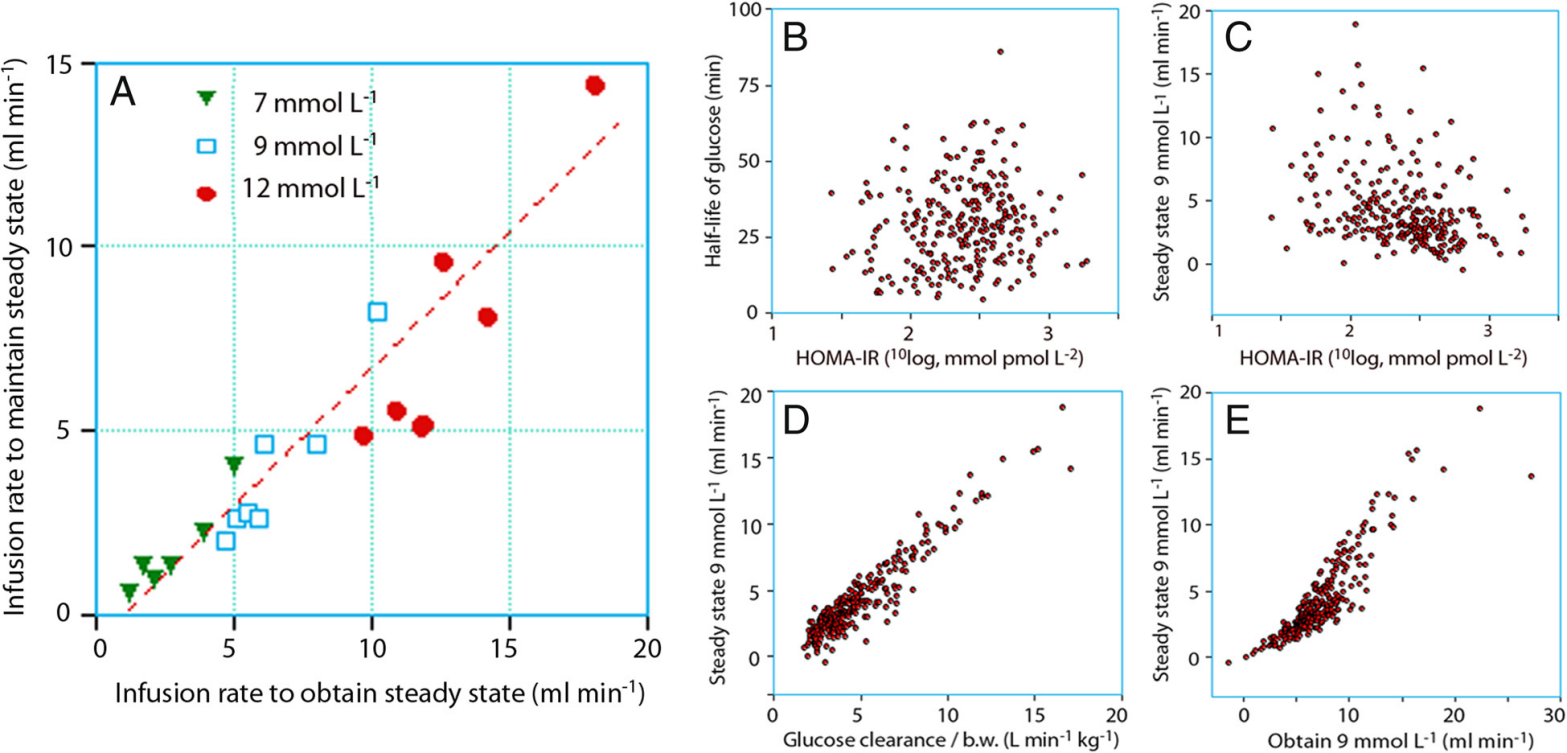
**a** Rates of infusion of glucose 5 % required to obtain a glucose concentration of 7, 9, and 12 mmol/l and the rates required to maintain this plasma concentration. Each data point is the mean value for one of the groups shown in Table 2. **b**, **c** Poor correlation between HOMA-IR and the disposition of glucose when infused intravenously. **d**, **e** Variability in the relationship between the CL and recommended rates of infusion. In subplots B to E, each point represents one infusion experiment

The infusion rates required to reach 7, 9, and 12 mmol/l within 30 min are shown in Table 2, right. After 30 min, each concentration can be maintained by reducing the rate of infusion to the one shown in Table 2, left. In all groups, the appropriate reduction is to 50–70 % of the rates needed to obtain the concentration (Fig. 3a).

HOMA-IR correlated poorly with the kinetic parameters obtained during the infusion experiments and also with the simulated maximum infusion rates (Fig. 3b–e).

## Discussion

Limits for the infusion rate of intravenous glucose are warranted, as studies demonstrate that, otherwise, patients are at risk of becoming markedly hyperglycemic (Sieber et al. 1987; Doze and White 1987). The consequences are dependent on the duration of the hyperglycemia and become worse in diabetics. Plasma glucose >10 mmol/l clearly increases the risk of postoperative infection, but there is also a higher likelihood of acute renal failure and death (Kwon et al. 2013; Frisch et al. 2010; Hanazaki et al. 2009; Lipshutz and Gropper 2009). Osmotic diuresis develops when plasma glucose is 12–15 mmol/l, which implies that the kidneys lose control of the fluid and electrolyte excretion.

The infusion rates suggested here are intended to be a guide for how to begin intravenous glucose therapy if hyperglycemia is to be avoided. They are calculated for glucose 5 %. Anesthetists who use glucose 2.5 % simply double the rates.

### Main results

The results show that the anesthetist has to consider at least a fourfold modification in infusion rate of glucose solution to account for the fact that hyperglycemia develops more easily in conjunction with surgery.

The infusion rates were clustered in three groups. The lowest were found during surgery and during the first hours after surgery (Table 2). Here, the calculated rates required to avoid plasma glucose >7 mmol/l were so low (0.5–1.5 ml/min) that providing glucose in this setting is hardly meaningful. A 1-l bag of glucose 5 % would need to be administered over 11–33 h to avoid hyperglycemia. In contrast, a rate of infusion of 2 ml/min would be possible if plasma glucose of 9 mmol/l was acceptable. That corresponds to an infusion time of 8 h for a 1-l bag.

The intermediate rates are found in the preoperative period and 2 days after surgery. Here, plasma glucose of 7 mmol/l could still easily be exceeded, if 9 mmol/l would be acceptable, glucose could be infused twice as fast as during surgery (1 l over 4 h). Finally, glucose can apparently be given to healthy individuals at an even higher rate without causing hyperglycemia.

Another way to administer glucose is by using a two-step strategy consisting of a more rapid initial infusion after which the rate is reduced to maintain a predetermined steady-state concentration. In the present series of simulations, we used 30 min as a reasonable time period for glucose loading. Most of the study groups would require quite similar amounts of glucose to raise the plasma glucose level—approximately 2–3 ml/min to increase the concentration to 7 mmol/l within 30 min in a subject weighing 70 kg. Most of the variability in infusion rates depicted in Table 2, right, is due to differences in baseline glucose, while between-patient differences in glucose kinetics become more apparent when attempting to maintain steady state.

A practical question is at which point in time a control blood sample should be taken when a check for hyperglycemia is desired in an individual patient. Such information is possible to derive from the half-lives given in Table 1. For a continuous infusion, half of the increase in plasma glucose takes place after *one* half-life, which is roughly 30 min in less stressful surgery such as laparoscopic cholecystectomy. As the final steady state takes about four half-lives to reach, the clinician would have to wait as long as 2–2.5 h until the maximum plasma glucose concentration is obtained. Therefore, a useful approach would be to take a control sample after 1 h, which roughly corresponds to two half-lives in the perioperative setting, and to consider that then the plasma glucose will increase by another 25 % if no adjustment to the infusion rate is made. This simple rule does not seem to be useful during open abdominal surgery as the half-life is much longer and also associated with greater variability.

### Limitations due to the studied cohorts

The patients included in this report are representative for a large proportion of the surgical population, while the suitable infusion rates may possibly be different in younger subjects and in special conditions. The studied patients were all in good health, which is an ethical requirement since the protocols involved volume loading that expanded the plasma volume by 10–15 %. No previous study has been performed which suggests suitable initial infusion rates for glucose in these groups of patients. The lack is probably explained by a well-spread belief that each patient must be evaluated individually because the plasma glucose responses to glucose infusions vary too much. Therefore, the existing literature offers surprisingly little guidance on this topic. However, the cohorts shown in Fig. 1 illustrate that the between-patient variation in plasma glucose is quite small in the perioperative period and that very good predictions can be made by taking the modeled average parameter values in a one-compartment model (thick blue lines) represent the individual plasma concentrations (thin red lines).

Open abdominal surgery was the only exception to this rule. The marked variability in the plasma glucose responses to exogenous glucose in this group could possibly be explained by variable efficiency of the thoracic epidural anesthesia to block the trauma response. In any event, repeated measurements of plasma glucose are warranted to guide glucose therapy if needed during this type of surgery.

Patients with diabetes, sepsis, and steroid treatment also need more individualized glucose administration and monitoring. Naturally, non-diabetic patients who receive intravenous insulin require much larger amounts of glucose than indicated here (Berndtson et al. 2008). The glucose metabolism shows that circadian rhythm responses may be greater to feeding during the dark period of the day (Kalsbeek et al. 2014).

### Insulin resistance and HOMA-IR

Insulin resistance is the key mechanism for the slowing of glucose turnover during and after surgery (Ljunggren et al. 2014a). Inactivity-induced impairment of cardiorespiratory fitness can induce some degree of insulin resistance even before surgery (Larsen et al. 2012). Another mechanism that raises plasma glucose is increased gluconeogenesis caused by psychological stress and surgical trauma. In our kinetic model, the summary effect of all these factors consists in a reduction of CL and a slightly raised plasma glucose concentration at baseline (Table 1).

The HOMA-IR did not reveal great differences between the groups and was of little or no help as a guide to the choice of infusion rates that avoid hyperglycemia. The HOMA-IR indicates insulin resistance as measured by the hyperinsulinemic glucose clamp in the “unstressed” state (Borai et al. 2007). However, HOMA-IR reflects hepatic insulin resistance (Borai et al. 2007) and recent evidence shows an increase by only 3–4 % in response to surgery (Ljunggren and Hahn 2012; Ljunggren et al. 2014b). In contrast, both the CL and the glucose clamp are strongly influenced by peripheral (skeletal muscle) insulin resistance, which might be doubled (Ljunggren et al. 2014a). Therefore, HOMA-IR only reflects the insulin resistance before surgery is undertaken and can be taken as an index of the effect of age and poor cardiorespiratory fitness on the glucose disposal. The studied patient groups varied in age, but only the volunteers (mean age 29 years) had a markedly lower insulin resistance than the others (Table 1). The disappearance rate of glucose is 10–15 % higher after a test meal in young as compared to old men and women, a difference that can be related mostly to insulin resistance (Basu et al. 2006). The incidence of diabetes type 2 also increases with age, but no such patients were included in this compilation.

### The kinetic model

The one-compartment kinetic model used here has been criticized for being simplistic and overlooking the endogenous glucose production, but it still offered excellent curve fits in individual subjects during the vast majority of the time period required for the glucose disposal. One of the downsides is that higher than modeled plasma glucose levels sometimes occur during the first minutes after a bolus infusion (Fig. 1c). This short “overshoot” can be studied by multi-compartment modeling (Ferranini et al. 1985) but is of limited relevance to the anesthetist who provides glucose as a continuous infusion at a low speed. Another issue is that slight hypoglycemia often develops 30–45 min after a glucose infusion given to a subject with high insulin sensitivity (Fig. 1a, d). This “undershoot” was not considered here and can be avoided clinically by gradual reduction of the infusion rate.

The one-compartment model is linear for glucose (Sjöstrand and Hahn 2003), which means that the predicted plasma glucose concentrations become similar regardless of whether the *V*_d_ and CL used for the simulation are derived from experiments providing small or large amounts of glucose, and regardless of whether the infusion time is long or short. In this report, *V*_d_ was larger for a 1-min injection compared to infusions, but the half-life of the glucose was still quite similar regardless of the infusion time (Table 1).

## Conclusions

Computer simulations based on kinetic data from seven studies suggested infusion rates that should be avoided to limit the risk of hyperglycemia in the perioperative period. Healthy volunteers can take infusion rates at least four times higher than patients on the day of surgery, and preoperative patients can take infusion rates twice as high. On the day of surgery, the acceptable rates are so low that infusing a glucose solution is hardly meaningful. Only very small amounts of glucose and free water can be provided at this time without imposing a risk of hyperglycemia.

## Appendix

A single-compartment kinetic model analyzed all plasma glucose data. Here, the relationship between the rate of infusion *R*_o_, the volume of distribution *V*_d_, and clearance CL was calculated using the following differential equation (Sjöstrand and Hahn 2003):

1 $$ \frac{d\left(C-{C}_b\right)}{dt}=\frac{R_{\mathrm{o}}}{V_{\mathrm{d}}}-\frac{CL}{V_{\mathrm{d}}}\left(C(t)-{C}_b\right) $$

where *C* is the plasma glucose at time *t* and *C*_b_ is the baseline level. The solutions to this differential equation before Eq. 2 and after Eq. 3 infusion are as follows:

2 $$ \left(C(t)-{C}_b\right)=\frac{R_o}{CL}\left(1-{e}^{-CL\times t/{V}_d}\right) $$

3 $$ \left(C(t)-{C}_b\right)=\frac{R_{\mathrm{o}}}{CL}\left(1-{e}^{-CL\times T/{V}_d}\right){e}^{-CL\times \left(t-T\right)/{V}_d} $$

where *T* is the infusion time. The optimal values of *V*_d_ and CL were estimated for each experiment separately via a Gauss-Newton least-squares regression routine applied to Eqs. 2 and 3, which had been entered into the MATLAB computer program.

In the same MATLAB environment, calculations were used to simulate the expected plasma glucose concentration at any time *t* in experiments not performed. The infusion rate (*R*_o_) needed to maintain a steady-state concentration (*C*_ss_) was obtained as

4 $$ {R}_o=\mathrm{C}\mathrm{L}\times \mathrm{B}\mathrm{W}\times \left({C}_{\mathrm{ss}}-{C}_{\mathrm{b}}\right)\times 0.0036 $$

where BW is the body weight (as CL is expressed per kilo body weight), *C*_b_ is the baseline glucose concentration for a group or individual, while 0.0036 converts mmol/min to ml/min of a glucose 5 % solution (the molar weight for glucose of 180 g is divided by 50 mg, which is the content of 1 ml of glucose 5 %).

The infusion rate required to reach a pre-determined plasma concentration *C*(*t*) within a certain time *t* was obtained as follows (in this study, *t* was set to 30 min):

5 $$ {R}_o=\frac{\mathrm{CL}\times \mathrm{B}\mathrm{W}\left(C(t)-{C}_b\right)\times 0.0036}{\left(1-{e}^{-\mathrm{C}\mathrm{L}\times t/{V}_d}\right)} $$

Equation 5 is a re-arrangement of Eq. 2 where *t* is set to 30 min. Equation 4, in turn, is a special case of Eq. 5 where the expression in the denominator approaches 1.0.

## Abbreviations

CL: clearance
HOMA-IR: homeostatic model assessment of insulin resistance
*V*_d_: volume of distribution
